# Human placenta-derived mesenchymal stem cells ameliorate orbital adipogenesis in female mice models of Graves’ ophthalmopathy

**DOI:** 10.1186/s13287-019-1348-0

**Published:** 2019-08-09

**Authors:** Mira Park, J. Paul Banga, Gi Jin Kim, MinYoung Kim, Helen Lew

**Affiliations:** 1 Department of Ophthalmology, CHA Bundang Medical Center, CHA University, Seongnam, Gyeonggi-do Republic of Korea; 2 Faculty of Life Sciences & Medicine, King’s College London, London, SE5 9NU UK; 3 Department of Biomedical Science, CHA University, Seongnam, Gyeonggi-do Republic of Korea; 4 Department of Rehabilitation Medicine, CHA Bundang Medical Center, CHA University, Seongnam, 13496 Gyeonggi-do Republic of Korea

**Keywords:** Graves’ ophthalmopathy, Graves’ disease, GO animal model, hPMSCs, Thyroid disease, Adipogenesis

## Abstract

**Background:**

Graves’ ophthalmopathy (GO) is a complication of Graves’ disease (GD), in which orbital connective tissues become inflamed and increase in volume and orbital fibroblasts within the orbital fat and extraocular muscles differentiate into adipocytes in vitro when stimulated by hormones, several cytokines, and growth factors including TSH, IGF-1, IL-1, interferon γ, and platelet-derived growth factor. Human placental mesenchymal stem cells (hPMSCs) have immunomodulatory effects in disease pathogenesis. Although a number of studies have reported that hPMSCs can elicit therapeutic effects, these are not sufficient. Therefore, we constructed a GO animal model in order to find out the hPMSCs recovery effect.

**Methods:**

We investigated their anti-adipogenic effects in in vitro cultures of orbital fibroblasts established from GO patients. Primary orbital fibroblasts were exposed to differentiation medium for 10 days. After being co-cultured with hPMSCs, the characteristics of orbital fibroblast were determined by Oil Red O stain and real-time PCR. Then, we explored the in vivo regulatory effects of hPMSCs in an experimental mouse model of GO. We developed the GO mouse model using immunization by leg muscle electroporation of pTriEx1.1Neo-hTSHR A-subunit plasmid. Human PMSC injection was performed into the left orbit. We also analyzed the effects of hPMSCs in the GO animal model.

**Result:**

We found that hPMSCs inhibited a lipid accumulation and activated factors, such as *ADIPONECTIN*, *PPARγ*, *C/EBPα*, and *TGFβ2* genes in adipogenesis-induced primary orbital fibroblasts from GO patients. Moreover, hPMSCs were highly effective at ameliorating adipogenesis in the orbital tissue of the model.

**Conclusion:**

These data indicate that hPMSCs recover pathogenic activation of orbital fibroblasts in animals undergoing experimental GO and confirm the feasibility of applying hPMSCs as a novel treatment for GO patients.

## Background

Graves’ disease (GD) is an autoantibody-induced immune disorder related to the thyroid-stimulating hormone receptor (TSHR) in the thyroid gland, causing toxic goiter or hyperthyroidism [1]. Graves’ ophthalmopathy (GO) is an autoimmune inflammatory disorder in which TSHR-stimulating antibody and TSHR influence several different cells in the periorbital tissue [2]. GO patients see the medical doctors for the thyroid eye manifestations as the first noticeable warning signs at the onset of the disease; otherwise, they could complicate the progression of the disease [3]. The typical thyroid eye features include proptosis, eyelid retraction, exposure keratopathy, restrictive strabismus, limitation of eye movement, compressive optic neuropathy, and appearance disfigurement. T cell infiltration and fibroblastic glycosaminoglycan accumulation are the main pathologies of the autoantibody-mediated inflammatory and fibrotic events in the periorbital fat and extraocular muscles (EOMs). Edematous swelling of the EOMs and adipogenesis of orbital fat lead to an increase of the volume within the bony orbital pyramid, and the common GO symptoms are followed such as irritation, orbital pain, tearing, diplopia, vision loss, corneal ulceration, and even blindness [4]. The major medical treatment methods to modify the disease course are high-dose steroids and radiation therapy. High-dose corticosteroids are effective in about 65% of patients. However, the total amount of corticoid use is restricted for the systemic adverse effects, including hyperglycemia, hypertension, and immune system compromise, and the eye symptoms usually recur when the steroid treatment is tapered or withdrawn [4–6]. Radiation therapy is another popular modality for the treatment of GO and is considered to treat the patients who are resistant to or cannot tolerate the side effects of corticoids. But the side effects of radiation therapy should also be kept in mind such as keratitis sicca, cataracts, and retinopathy [7, 8]. A number of advanced therapies have been introduced, most of which have studied about immunomodulatory effects; these include rituximab (RTX), tocilizumab (TCZ), the humanized anti-interleukin-6 receptor monoclonal antibody, teprotumumab, IGF-1 receptor-blocking antibodies, and TNF-α inhibitors [9, 10].

Human placental mesenchymal stem cells (hPMSCs) are the cells with self-renewing abilities originated from human placenta that can differentiate into multiple lineage cell types. Various studies have reported that hPMSCs can elicit therapeutic effects via differentiation and/or secretion of factors such as growth factors, cytokines, and chemokines [11]. hPMSCs contribute to the repair of tissue damage caused by ischemic diseases including strokes, myocardial infarctions, and cerebral infarctions [12–14]. Furthermore, in the field of ophthalmology, cryopreserved amniotic membranes and their by-products have been recognized as significant tools for the treatment of ulceration and epithelial defects over the past 20 years [15, 16]. Recently, a number of translational studies have reported that the anti-inflammatory effects of hPMCSs can be used to modulate chronic diseases such as Crohn’s disease, multiple sclerosis, and sarcoidosis [17–19]. In this study, we investigated the immunomodulatory effects of hPMSCs in the case of GO using both in vitro tests with orbital fibroblast (OF) cultures and in vivo tests and compared our results with those of conventional steroid treatments in an experimental mouse model of GO [20, 21].

## Materials and methods

### Orbital fibroblast preparation

Orbital adipose tissue explants were obtained from three GO patients during orbital fat decompression and three control individuals with no history of GO during blepharoplasty. The process of obtaining orbital adipose tissue was approved by the Institutional Review Board of Bundang CHA Medical Center (Seongnam-si, South Korea), and consent was obtained from all patients. Tissue explants were chopped and treated with collagenase (0.25 mg/mL; Thermo Fisher Scientific, Waltham, MA, USA) for 1 h at 37 °C in a shaking incubator. After digestion, the tissues were placed directly in culture dishes with DMEM/F12 containing 20% fetal bovine serum (FBS; Thermo Fisher Scientific) and 1% penicillin/streptomycin (Thermo Fisher Scientific). The cells were serially passaged, and the results of the fifth to eighth cell passages were used for the experiments.

### Human placenta stem cell preparation

We previously described the methods for preparing hPMSCs [22]. The collection and use of the samples were approved by the Institutional Review Board of CHA General Hospital (Seoul, South Korea). All of the participants provided written informed consent prior to the sample collection. Briefly, the placentas were collected from mothers who were free of medical, obstetric, and surgical complications who delivered at term (> 37 gestational weeks). The hPMSCs were collected from the inner side of the chorioamniotic membrane of the placenta and then treated with 0.5% collagenase IV (Sigma-Aldrich, St. Louis, MO, USA). Human PMSCs were cultured in Minimum Essential Media (MEM) alpha GlutaMAX (Thermo Fisher Scientific) supplemented with 10% FBS (Thermo Fisher Scientific), 1% penicillin/streptomycin (Thermo Fisher Scientific), 25 ng/mL human fibroblast growth factor 4 (Peprotech, Inc., Rocky Hill, NJ, USA), and 1 μg/mL heparin (Sigma-Aldrich).

### Adipocyte differentiation

Human orbital fibroblasts (hOFs) (3 × 10^5^) derived from either fresh cultures or frozen cells were seeded in a 6-well plate and incubated until they reached the desired confluence. For all experiments, OFs derived from GO patients were grown together with OFs from normal individuals as controls. To obtain confluent cultures, adipogenesis was induced by replacing the culture medium with DMEM supplemented with 10% FBS, 1% penicillin/streptomycin, 33 μM biotin, 17 μM pantothenic acid, 0.2 nM T3, 10 μg/mL transferrin, 0.2 μM prostaglandin I2, 0.1 mM isobutylmethylxanthine (IBMX), 1 μM dexamethasone, and 1 μM insulin (Sigma-Aldrich). The differentiation-induced medium was replaced every day for 4 days. Thereafter, the medium was changed to differentiation medium lacking IBMX, dexamethasone, and insulin, and the cells were cultured for up to 8 days more, after which Transwell cell culture plate insert was put on 6-well plate. Then, hPMSCs (3 × 10^5^) were seeded into the insert membrane. After 48 h, the co-cultures of OFs and hPMSCs were used for experimentation, as described below.

### Oil Red O staining

After 10 days of adipocyte differentiation, cells were stained with Oil Red O. The working solution was prepared by diluting 6 mL stock solution (0.5% Oil Red O in isopropanol) in 4 mL distilled water. After washing with DPBS, cells were fixed in 10% formaldehyde solution for 1 h at room temperature (RT). After fixation, the cells were washed with distilled water and stained with Oil Red O solution for 30 min at RT. After washing with distilled water, stained cells were visualized under a microscope. To quantify lipid accumulation, 100% isopropanol was added into the stained well. Then, the optical density was measured using a spectrophotometer at 490 nm.

### Quantitative reverse transcription polymerase chain reaction analyses

Total RNA was isolated from hOFs using TRIzol reagent (Ambion, Carlsbad, CA, USA). Quantitative real-time PCR (qPCR) was performed with IQ SYBR Green Supermix (Bio-Rad Laboratories, Hercules, CA, USA). We quantified the gene expression using the delta CT method, and qPCR reactions were performed using a CFX-96 machine (Bio-Rad Laboratories). The nucleotide sequences of all primers used are presented in Table 1.

**Table 1:** Sequences of primers

Gene	Sequences (5′ → 3′)
ADIPONECTIN	F: GGCCGTGATGGCAGAGAT
	R: TTTCACCGATGTCTCCCTTAGG
PPARγ	F: TTGACCCAGAAAGCGATTCC
	R: AAAGTTGGTGGGCCAGAATG
C/EBPα	F: TGTATACCCCTGGTGGGAGA
	R: TCATAACTCCGGTCCCTCTG
C/EBPβ	F: CTTCAGCCCGTACCTGGAG
	R: GGAGAGGAAGTCGTGGTGC
TGFβ2	F: TGGTGAAAGCAGAGTTCAGAG
	R: CACAACTTTGCTGTCGATGTAG
CSH1	F: AAACTCGCACAACCATGACG
	R: GAGGGGTCACAGGATGCTACTC
18 s rRNA	F: TGAGAAACGGCTACCACATC
	R: ACTACGAGCTTTTTAACTGC

### Development of an experimental mouse model of GO using female BALB/c mice

We developed a GO mouse model using immunization by leg muscle electroporation of pTriEx1.1Neo-hTSHR A-subunit plasmid as previously described [21]. BALB/c female mice (age 6 weeks) (Orient Bio Inc., Gyeonggi-do, South Korea) were housed in a standard animal facility, with food and water provided ad libitum at a constant temperature of 21 °C. The animals were maintained in an air-conditioned animal house under specific pathogen-free conditions. The animal protocol was approved by the Institutional Animal Care and Use Committee of CHA Bundang Medical Center. We injected 50 μL plasmid (1 mg/mL in BSS PLUS Irrigating Solution) into the leg muscles of BALB/c female mice (age 6 weeks), followed by electroporation using an ECM 830 system (BTX Harvard Apparatus, Holliston, MA, USA). Electroporation was performed at 200 V/CM and 10–20 ms square wave pulses. Injection and in vivo electroporation were performed four times, at 3-week intervals. After the 13-week immunization period, we injected the mice with BSS, hPMSCs, and steroids.

### Immunomodulation

**Fig. 1:**
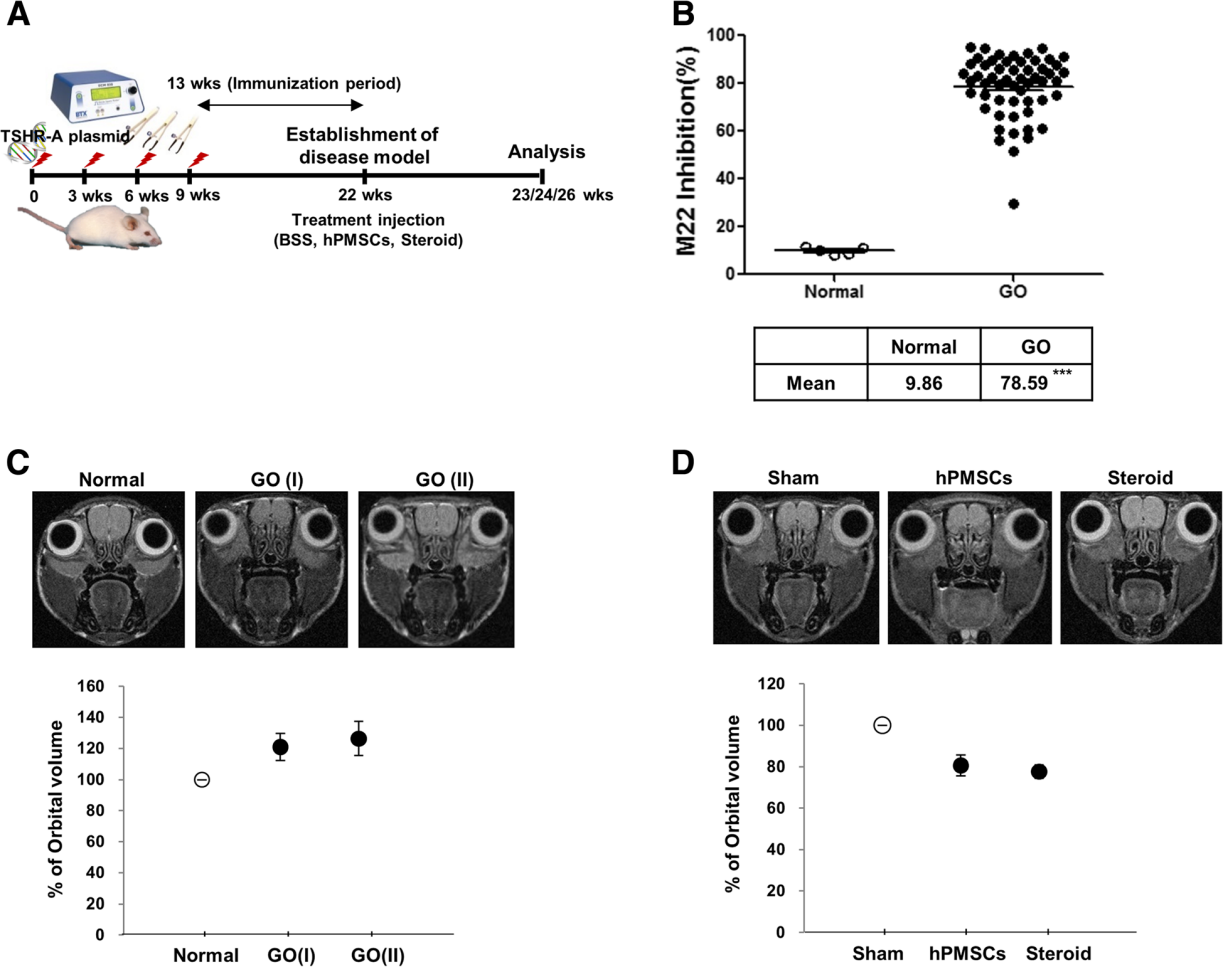
Characterization studies on hTSHR A-subunit plasmid immunized mice. **a** Scheme of GO disease model construction. **b** Anti-TSHR Ab inhibition in the serum of disease mice at 12 weeks after last immunization with pTriEx-1.1 TSH receptor (TSHR) A-subunit plasmid in the muscle combined with electroporation. The serums from 5 normal animals and 55 GO disease animals were used in the analysis. Frontal MRI analysis shows protrusion of the eye and hypertrophy of the orbital volume shown as EOM area from **c** an age-matched normal and GO disease mice. In GO disease model, **d** the typical MRI analysis from BSS- (sham), hPMSC-, or steroid-injected groups after 4 weeks was presented. Significantly different values between the groups are indicated with asterisks (***p < 0.0005)

At 22 weeks after the first immunization (Fig. 1a), all of the immune animals were bled to evaluate the induced anti-TSHR antibodies. The hyperthyroid animals undergoing experimental GO were divided into the following groups: a treatment group injected with hPMSCs (*n* = 14; 3 × 10^5^ cells/30 μL), a treatment group injected with steroids (*n* = 14; 0.4 mg each, triamcinolone acetonide, Dongkwang Pharmaceutical Co., Hanmi, South Korea), and a sham group (*n* = 12; 30 μL BSS PLUS). An intra-orbital injection was performed into the left orbit. After single hPMSC injection, the animals were sacrificed after 1, 2, or 4 weeks, after which their blood was collected for serum, and orbital tissue was excised for histopathological analyses.

### Measurement of antibodies to TSHR

We confirmed that GO animal models had been achieved before dividing the mice into treatment groups based on the analyses of their serum at 21 weeks using the anti-TSH receptor antibody (TRAb) from the Fast ELISA commercial kit (EUROIMMUN, Luebeck, Germany). We used the antibodies as indicated by the manufacturer’s instructions. The results are expressed as the percentage inhibition of labeled M22 binding to the immobilized TSHR in the plate wells.

### Measurement of cytokines in the blood

The levels of pro- and anti-inflammatory cytokines in the serum of immune mice were determined using the Magnetic Luminex® Screening Assay (Thermo Fisher Scientific), according to the manufacturer’s instructions. The values were measured using Luminex 100 (Luminex, Austin, TX, USA). The following cytokines were measured: IL-1β, IL-2, IL-4, IL-6, IL-10, TNFα, ICAM-1, G-SCF, and GM-CSF.

### In vivo orbital magnetic resonance imaging

Immune animals with GO (*n* = 2) and age-matched normal mice (*n* = 1) were evaluated via magnetic resonance imaging (MRI) using the 4.7 T MRI system (BioSpec 47/40; Bruker, Ettlingen, Germany) at the Korea Basic Science Institute. To acquire in vivo MR images, the animals were anesthetized with inhaled isoflurane (5% induction, 1.5% maintenance) at a O_2_–N_2_O (3:7) mixture, and their body temperatures were maintained at 37 ± 1 °C. Anesthesia was induced and maintained by administering a 1–2% isoflurane–oxygen mix throughout the imaging process. The head of each mouse studied was situated within an MRI volume coil with an internal diameter of 25 mm. Axial T2-weighted MR images were acquired using a rapid acquisition with relaxation enhancement (RARE) sequence with the following settings: repetition time (TR), 4 s; effective echo time (TE), 33 ms; echo train length, 8; field of view (FOV), 20 mm × 20 mm; and matrix size, 256 × 256 (50 μm in-plane resolution). The ImageJ software application (NIH) was used to view the MR images and measure the orbital volume of the left eye. After confirming the regional volume differences between the controls and GO models via MRI, another three GO animals were treated with hPMSCs, steroids, or injected BSS. Four weeks later, we conducted MRI analyses of these immunized mice.

### Orbital and thyroid tissue histopathology

Quantification of the adipose tissue around the optic nerve was conducted using Image J. The cross-sectional area of the orbital fat was normalized to the optic nerve area of each mouse. The adipose areas of the orbital sections of each mouse were evaluated in every group.

### Immunoblot analyses of target proteins and cell signaling pathways

Lysates were prepared from orbital tissues, which were injected with BSS (sham), hPMSCs, or steroids by homogenization with PRO-PREP solution (Intron, Gyeonggi-do, South Korea). Equal amounts of total protein were resolved by sodium dodecyl sulfate-polyacrylamide gel electrophoresis (SDS-PAGE) and transferred to the membranes. The membranes were immunoblotted with anti-ICAM-1 (Thermo Fisher Scientific), TGFβ1, TGFβ2 (GeneTex, Irvine, CA, USA), TNF-α (Thermo Fisher Scientific), TSHR (NSJ Bioreagents, San Diego, CA, USA), phosphorylated ERK (p-ERK), total ERK, AKT (Cell Signaling Technology, Denver, MA, USA), mTOR (Thermo Fisher Scientific), or α-tubulin (GeneTex). After washing, the membranes were incubated at RT for 2 h with horseradish peroxidase-conjugated anti-rabbit or mouse IgG secondary antibodies at a dilution of 1:5000 (GeneTex). Immunoreactive bands were visualized with enhanced chemiluminescence solution (Bio-Rad Laboratories, Hercules, CA, USA) and detected using an ImageQuant LAS 4000 (GE Healthcare Life Sciences, Little Chalfont, UK).

### Engraftment of hPMSCs

We confirmed the presence of injected hPMSCs in orbital tissues by analyzing them in two ways. (1) The tissues from each group were embedded in OCT compound. After making slide sections, DAPI staining was performed. Fluorescence was detected using a fluorescence microscope. (2) cDNA was synthesized using orbital tissues. Expression of the human-specific gene *chorionic somatomammotropin hormone 1* (*CSH1*) was detected in cDNA extracted from tissues injected with hPMSCs. The primers used are presented in Table 1.

### Statistical analyses

Data analyses were conducted using GraphPad Prism (GraphPad Software, La Jolla, CA, USA). Statistically significant differences were identified using a *t* test or nonparametric statistical test, followed by Mann-Whitney *U* test at a significance level of 5%.

## Results

### Characterization studies

To investigate the therapeutic function of hPMSCs, we established an experimental mouse model of GO as described in a recent study [20]. Human TSHR A-subunit plasmid was delivered to mice by electroporation (Fig. 1a). In a TSH-binding inhibitory immunoglobulin (TBII) assay using an anti-TSH receptor (TRAb) ELISA, the GO models exhibited > 78% inhibition of labeled TSH binding activity compared to the control group (Fig. 1b). The orbital region of the animals was examined using small animal MRI and was compared to the orbital region of age-matched control mice. The orbital volumes measured from the MR images increased by 20–30% compared to the age-matched controls (Fig. 1c). Human PMSCs decreased by > 20% in the orbital volume around the eyes of GO mice immunized with hTSHR A-subunit plasmid. Steroids also had a similar efficacy at reducing the orbital volume (Fig. 1d).

### Pathology assessment

**Fig. 2:**
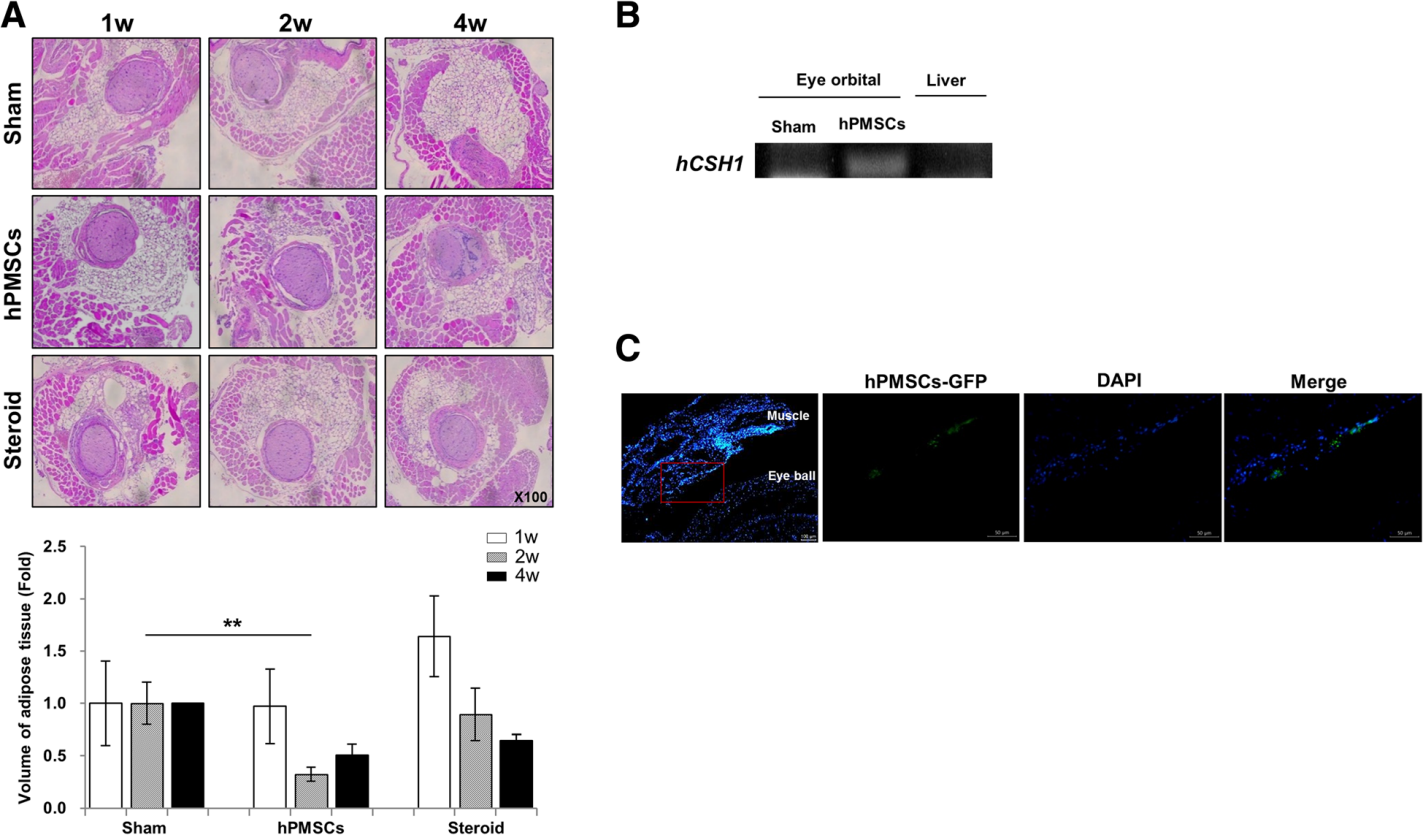
Histologic analysis of animals undergoing experimental GO treated with hPMSC or steroids. **a** H&E-stained section from GO mice with an expansion of retrobulbar adipose tissue around the optic nerve (× 100). Using **b** cDNA from mice orbital tissues and liver and **c** OCT-embedded mice tissues, the presence of hPMSCs was found between the extraocular muscle and eyeball. Data was presented as the fold changes (means ± SEM) of adipose volume compared with the sham of each group. Significantly different values between the groups are indicated with asterisks (**p < 0.01)

Next, we assessed the pathology of the GO groups. We used hemotoxylin and eosin staining to measure the volume of orbital tissues in each group. At 2 weeks post-transplantation, the retrobulbar adipose tissue of the hPMSC-injected mice showed a significant reduction in the pathologic expansion (Fig. 2a). Furthermore, although disease expansion also decreased in tissues injected with hPMSCs and steroids, the reductions were not significant (Fig. 2a).

### Engraftment of hPMSCs

We confirmed the infiltration of injected hPMSCs by assessing the eye orbital tissues and liver samples. We confirmed the mRNA expression of *human CSH1*, which is highly expressed in the placenta. Hence, the hPMSCs remained successfully engrafted by our procedure (Fig. 2b). As a negative control, we could not find *hCSH1* band in liver tissues (Fig. 2b). We also observed PKH67-labeled green fluorescent cells in local regions surrounding the sites injected with hPMSCs at 1 week, further supporting the successful engraftment of stem cells (Fig. 2c).

### Human PMSCs attenuate pro-inflammatory cytokines

**Fig. 3:**
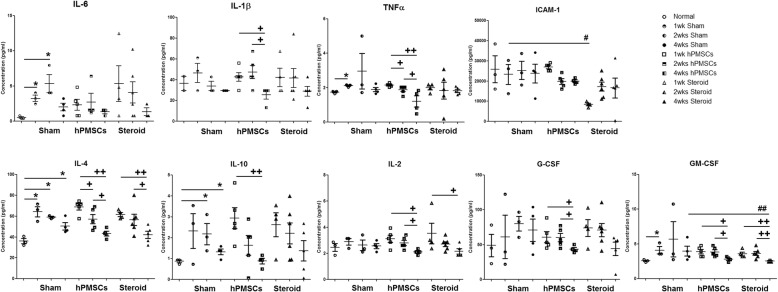
Cytokine expression profiles among the sham versus treatment groups in GO animal models and normal mice. Total nine cytokines were examined through Luminex bead array. Significantly different values between the groups are indicated with marks (*p < 0.05 vs normal mice; ^#^*p* < 0.05, ^*##*^*p* < 0.005 vs age-matched sham; ^**+**^*p* < 0.05, ^**++**^*p* < 0.01 vs intra-treatment groups). At least three animals of each group are used

We also investigated whether hPMSCs could reduce the inflammatory response in GO disease models by measuring their concentration in serum samples. As shown in Fig. 3, sham GOs presented a significantly higher level of various anti- and pro-inflammatory cytokines than normal mice, IL-6, TNFα, IL-4, and GM-CSF at 1 week; IL-6, IL-4, IL-4, and IL-10 at 2 weeks; and IL-4 and IL-10 at 4 weeks. Compared with the sham GOs, serum from hPMSC-injected GO models contained similar levels of cytokines with age-matched sham, but that from steroid-injected GO presented reduced level of ICAM-1 at 1 week and GM-CSF at 2 weeks and 4 weeks (Fig. 3).

### Human PMSC transplantation decreases candidate target protein expression in the GO animal model

**Fig. 4:**
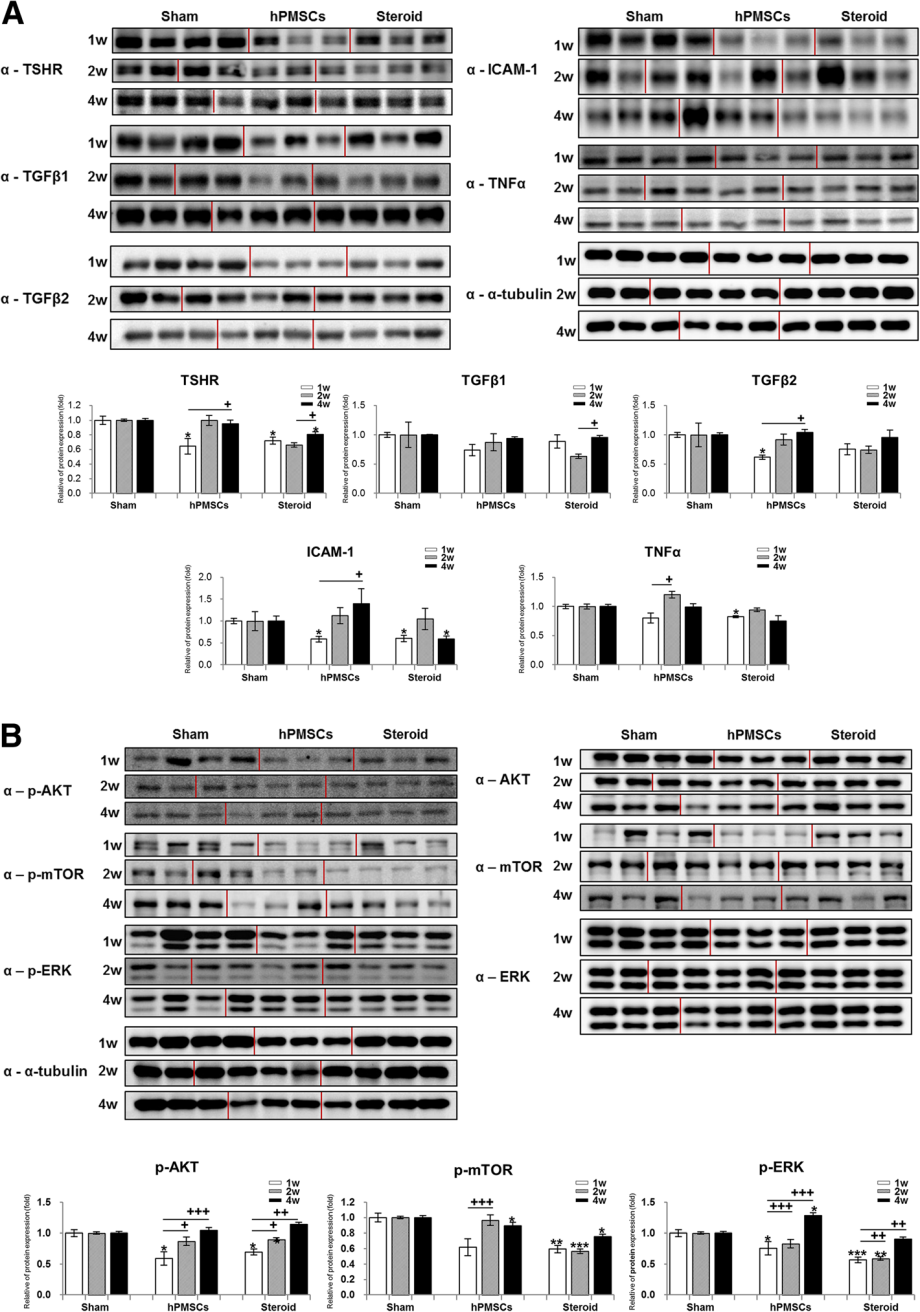
Human PMSCs attenuated target proteins level in GO animal model. The resultant changes in **a** TSH-receptor, TGFβ1, TGFβ2, ICAM-1, and TNF-α and **b** general signaling involved proteins were examined by immunoblot analysis of GO mice orbital tissues. Expression levels were normalized to α-tubulin, and the quantified values of target proteins expression are also presented (down panel). Significantly different values between the groups are indicated with marks (*p < 0.05, **p < 0.005, ***p < 0.001 vs age-matched sham; ^**+**^*p* < 0.05, ^**++**^*p* < 0.01, ^**+++**^*p* < 0.001 vs intra-treatment groups). The red lines on the immunoblot data presented each group

We investigated the target protein expression in the orbital tissues of the GO animal models. The expressions of TSHR, TGFβ2, ICAM-1, and TNFα significantly decreased following treatment with hPMSC or steroid injections. Specifically, hPMSCs significantly reduced the levels of the target protein at 1 week post-transplantation. Steroids affected TSHR and ICAM-1 up to 4 weeks post-transplantation (Fig. 4a).

### Human PMSCs attenuate target protein levels in GO animal models via the p-AKT and ERK signaling pathways

As shown in Fig. 4a, hPMSCs reduced the levels of some target proteins. Thus, next, we investigated the signaling pathways involved in regulating the target proteins by assessing the general signal relative to protein expression. We observed less p-AKT, p-mTOR, and p-ERK expression in the treatment injection groups (Fig. 4b). Moreover, steroids inhibited the activation of p-AKT, p-mTOR, and p-ERK longer than hPMSCs (Fig. 4b).

### Human PMSCs inhibit lipid accumulation by regulating adipogenic factors

**Fig. 5:**
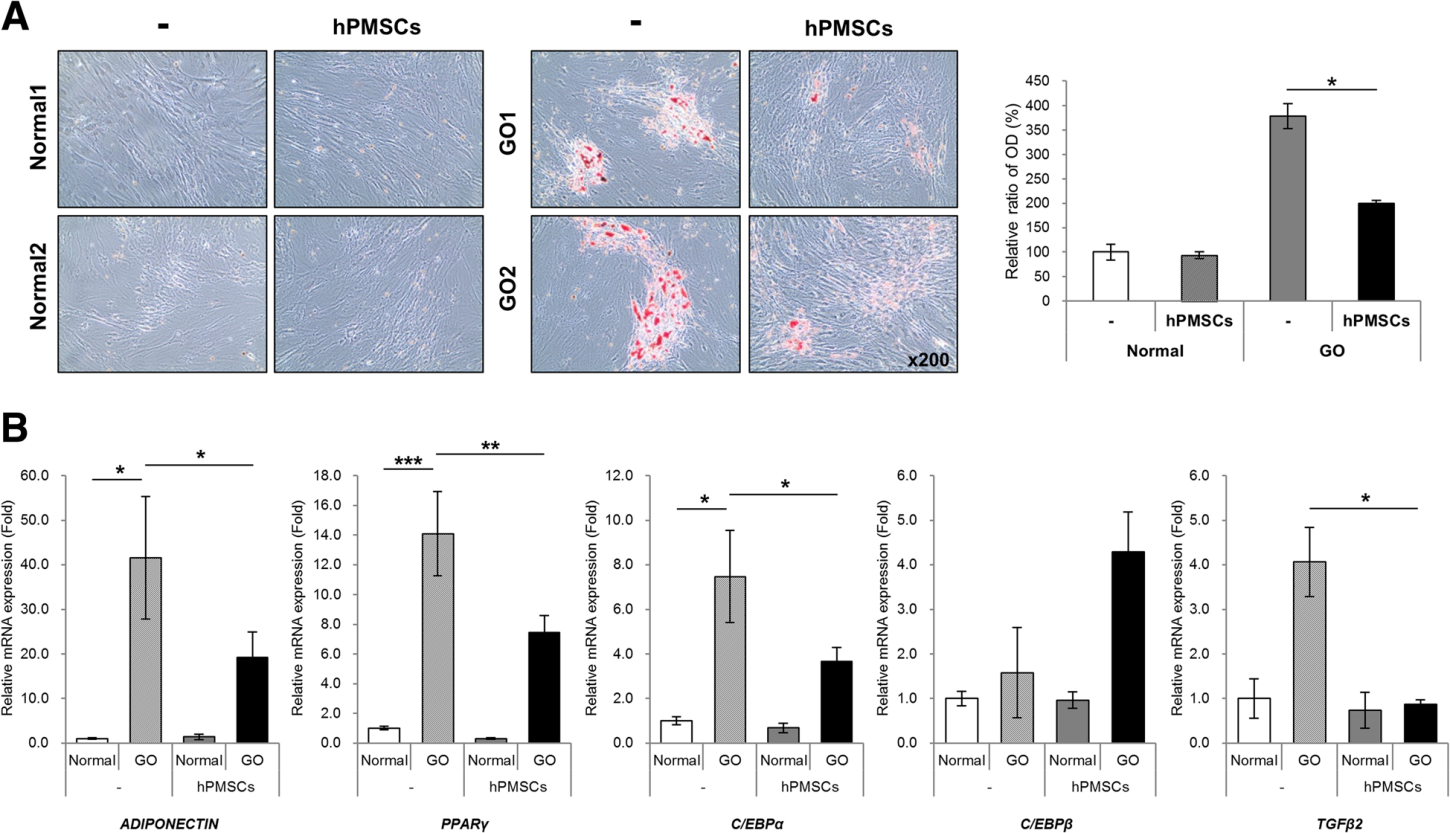
Human PMSCs have effects on adipogenesis of orbital fibroblasts. The orbital fibroblasts from normal and GO patients were co-cultured with hPMSCs during 2 days of adipose differentiation. After 10 days, **a** cells were stained with Oil Red O solution or **b** cell lysates were subjected to mRNA expression analysis to determine *ADIPONECTIN*, *PPARγ*, *C/EBPα*, and *TGFβ2* genes. Significantly different values between the groups are indicated with asterisks (*p < 0.05, **p < 0.005, ***p < 0.0005)

The effects of hPMSCs on lipid accumulation in hOFs are presented in Fig. 5a. After adipocyte differentiation, we observed Oil Red O-stained adipocytes in OFs collected from GO patients and normal. Lipid accumulation in differentiated fibroblasts was decreased by co-culturing with hPMSCs. To understand the molecular mechanisms underlying the inhibition of lipid accumulation, we evaluated the gene expression involved in lipid biosynthesis. The effects of hPMSCs on the differentiation of adipose fibroblasts were investigated by qPCR analyses (Fig. 5b). As expected, in fibroblasts co-cultured with hPMSCs, *ADIPONECTIN*, *PPARγ*, *C/EBPα*, and *TGFβ2* mRNA levels were significantly decreased compared to the cells that were not co-cultured. This suggests that hPMSCs regulated adipogenic factors and clearly indicates that hPMSCs inhibited the accumulation of lipids in OFs from GO patients.

## Discussion

GO is a complication of GD, but mechanistic insights into its pathogenesis are lacking, which have been hampered by the lack of an animal model. The TSHR and perhaps IGF-1 receptor are considered to be relevant antigens [10, 23]. Deficient immune tolerance to THSR contributes to the development of GO [24]. The activation of T cells and autoantibodies to TSHRs are related to the pathogenesis of TAO [10, 24]; however, the details of this process remain unclear. GO is caused by an inflammatory process that increases the volume of extraocular muscle (EOM) and causes histological changes in orbital tissues [25]. Recently, to investigate the pathogenesis of GD, a number of studies based on mouse models have been reported [26, 27]. OFs from GO patients have also been characterized [28–30]. Several studies have provided experimental evidence that OFs derived from retrobulbar fat of GO patients have similar immunophenotypic features and differentiation abilities as MSCs but differ in terms of their immunosuppressive potential [28, 29]. Furthermore, comparative analyses of adipose-derived MSCs from GO orbital fat and abdominal fat have been conducted to understand the phenotypic characteristics of OFs in GO patients [30].

However, it is not sufficient to understand the complexities of the orbital remodeling process. In previous studies, we observed that genetic immunization by injection of human TSHR A-subunit plasmid led to the remodeling of orbital tissue, recapitulating the pathological features of GO [20, 21]. In those studies, orbital pathology was characterized in terms of the interstitial inflammation of EOMs with CD3+ T cells and F4/80+ macrophages, adipogenesis, and fibrosis. In addition, in vivo MRI scans of the orbital regions of mice have provided clear and quantifiable evidence of orbital muscle hypertrophy with protrusion (proptosis) of the eye [20]. MR imaging can be applied to diagnose GO and assess patient responses to treatment [31, 32]. GO animal models exhibit IL-10, IL-6, and TNF-α cytokine responses to activated T cells [21].

Despite the fact that there have been a number of studies on GO pathogenesis, there are not yet definite diagnostic criteria or therapeutic treatments for GO. A number of treatments for specific therapeutic targets have been reported. The applicability of a particular treatment for GO depends on the severity and activity of TAO. For patients with mild, active TAO, short courses of oral glucocorticoids and/or anti-inflammatory agents may be sufficient [10]. In the case of moderate to severe and active TAO, nonspecific anti-inflammatory agents are commonly used. However, systemic corticosteroids are administered in some cases [10]. Because glucocorticoids are associated with the risk for adverse events, various treatments have been developed for specific targets. Teprotumumab (a repurposed IFG-IR inhibitor), rituximab (anti-CD20), B cell-depleting agents, and tocilizumab may be candidate treatments for the treatment of advanced TAO [10, 32]. Furthermore, low-dose radiotherapy (LD-RT) has been considered an alternative to treatment with systemic anti-inflammatory reagents. This induces an inflammatory response in irradiated tissues, thus producing an anti-inflammatory effect [32]. The response to LD-RT is regulated by a sequence of interactions between leukocytes and the endothelium. The mechanisms governing this response include decreased leukocyte-endothelial cell adhesion, induction of cell death by inflammatory infiltration, and decreased expression of adhesion molecules (P-, L-, E-selectins, ICAM-1, VCAM-1) [32–34].

Three types of TSHR monoclonal antibody (stimulating, blocking, and cleavage antibodies) have different functional capabilities in GD patients [24]. M22, a widely used and high-affinity stimulating human mAb to TSHR, is considered an international standard [35]. Stimulating antibodies induce thyrocyte cell survival and proliferation via cAMP/PKA/CREB and Akt/mTOR/S6k signaling. On the contrary, cleavage antibodies result in apoptosis via ROS induction and NF-κb activation. The balance between negative and positive regulations may be important for maintaining thyrocyte homeostasis in GD [24].

In a previous study, we investigated that NF-κb could be the mediator protein in the hPMSC-related recovery pathway [36]. Transactivation of NF-κb protein would take an important part in the recovery process from hypoxic damage. We also found out that hPMSCs could upregulate the expression of axon survival-related genes and support recovery from optic nerve compression. This helped us understand the potential role of hPMSCs as a treatment modality for the traumatic optic nerve injury [36]. Immunomodulatory properties of cultured MSCs were discussed that they could directly suppress the proliferation of T cells in vitro [37–39] and also avoid cytotoxic T cell-mediated lysis [40]. Furthermore, they can both inhibit [41] and promote [42] B cell proliferation, hinder NK cell activation [43, 44], and control the secreted cytokines from dendritic cells [45] and macrophages [46]. Their collaboration with dendritic cells and macrophages provides the circulatory influence over the immune system. And the possible mediation of the suppressive effect of MSCs on B cells was studied that a CCL2 variant modified with matrix metalloproteinase has suppressed immunoglobulin production by plasma cells [47].

Recently, the dynamic expression of cytokines secreted from MSCs was reported to control T cell function and maturation by regulating the expression of FoxP3, prominently in T cell differentiation. Treg cells co-cultured with PMSCs significantly expanded in comparison with others with bone marrow-derived mesenchymal stem cells (BMSCs) [48]. On the other hand, BMSCs should be harvested by an invasive procedure, are uncommon in the adult human bone marrow [49], and their number consequently reduces with the age of the person [50]. But the placenta could be an abundant source of stem cells, no invasive procedures are required to gain the organ, and they are free from the ethical debate about their use as a source. The other advantages are that PMSCs have abilities of multi-lineage differentiation like BMSCs in the perspectives of morphology, cell surface antigen existence, and gene expression features; are capable of differentiation into various cell types; are easy to separate; and an abundant amount of MSCs can be taken in culture [51].

There are other molecules mediating the immunomodulatory events of MSCs such as IL-10, human leukocyte antigen G (HLA-G) [52–54], and leukemia inhibitory factor (LIF) [55], the latter of which plays a critical role in the regulation of T cell proliferation and production and preservation of Treg cells [56]. Cultured placenta-derived MSCs produced the immunomodulatory effects in vivo, although in most cases, describing the molecules accountable for the discovered immunomodulation was disturbed by the complex communication between MSCs and endogenous cells, which stimulated the host’s intrinsic immune system. Regarding the immune tolerance of the eye, Wang et al. performed the experiment of the intact amniotic epithelium grafts from allogeneic GFP+ mice with syngeneic (EGFP-C57BL/6 to C57BL/6 W/t) and allogeneic (EGFP-C57BL/6 to BALB/c W/t). The major histocompatibility (MHC) class II+ antigens were quite less expressed post-implantation, when amniotic epithelial cells were implanted on the anterior surface of the eye or inserted into the anterior chambers of the eye. [57]. Le Blanc et al. proposed that MSC infusions would be useful to treat severe patients with steroid refractory graft versus host disease [58].

## Conclusion

In conclusion, although analyses of various orbital pathologies have been reported, studies on the therapeutic effects of stem cells have been lacking. This is the first report on the therapeutic effects of stem cells on a GO animal model. Based on our findings, we suggest that hPMSCs have immunomodulatory effects, inhibiting adipogenesis via anti-inflammatory effects. These results suggest that hPMSCs could potentially be used as a new therapy for GO disease.

## Data Availability

All data and materials are available upon request.

## Abbreviations

GD: Graves’ disease
GO: Graves’ ophthalmopathy
hPMSCs: Human placenta-derived mesenchymal stem cells
TAO: Thyroid-associated ophthalmopathy
